# Clay-Based Polymer Nanocomposites: Essential Work of Fracture

**DOI:** 10.3390/polym13152399

**Published:** 2021-07-22

**Authors:** Edgar Adrian Franco-Urquiza

**Affiliations:** National Council for Science and Technology (CONACYT—CIDESI), Center for Engineering and Industrial Development, Carretera Estatal 200, km 23, Querétaro 76265, Mexico; edgar.franco@cidesi.edu.mx

**Keywords:** EWF, polymer-layered silicates, oMMT, clay minerals, mechanical properties

## Abstract

This work details the general structure of the clays used as a reinforcement phase in polymer nanocomposites. Clays are formed by the molecular arrangement of atomic planes described through diagrams to improve their visualization. The molecular knowledge of clays can facilitate the selection of the polymer matrix and achieve a suitable process to obtain clay-based polymer nanocomposite systems. This work highlights the development of polymer nanocomposites using the melt intercalation method. The essential work of fracture (EWF) technique has been used to characterize the fracture behavior of materials that show ductility and where complete yielding of the ligament region occurs before the crack propagation. In this sense, the EWF technique characterizes the post-yielding fracture mechanics, determining two parameters: the specific essential work of fracture (*w_e_*), related to the surface where the actual fracture process occurs, and the specific non-essential work of fracture (*w_p_*), related to the plastic work carried out in the outer zone of the fracture zone. The EWF technique has been used successfully in nano-reinforced polymers to study the influence of different variables on fracture behavior. In this work, the fundamentals of the EWF technique are described, and some examples of its application are compiled, presenting a summary of the most relevant contributions in recent years.

## 1. Introduction

Thermoplastic polymers are commonly reinforced with inorganic fillers to improve various properties and reduce costs. Conventional inorganic fillers include talc, calcium carbonate, silica, mica, among others. Achieving a significant improvement in the properties of the reinforced polymer often requires incorporating a relatively large amount of filler, which leads to a loss of transparency and increases the volume and density of the polymers. Depending on the dimensions of the phases involved, the particle-filled polymers are classified as microcomposites. However, the technological demand in innovation and materials engineering allows the constant development of polymers reinforced with inorganic particles to increase their mechanical resistance and integrate other functionalities such as thermal conductivity, heat capacity, thermal diffusion, and self-healing [1].

This review collects information on the influence of clay minerals on the fracture behavior of ductile nanocomposites, employing the essential work of fracture (EWF) approach and focusing on the process-properties relationship. There is extensive literature on the structure and characterization of clay minerals. However, studies on relevant aspects such as the arrangement of atomic planes, which are results of interest for the preparation of clay-based polymer nanocomposite systems, have not been published. On the other hand, the influence of clay minerals on the ductile fracture behavior of polymer nanocomposites has not been fully explored, and this is the gap that this review intends to cover.

Although vast amounts of information about clay-based polymer nanocomposite systems is available, this review aims to disseminate knowledge in a very didactic way. Therefore, it is addressed primarily at graduate students entering the exciting field of developing new clay-based polymer nanocomposite systems.

## 2. Clay-Based Polymer Nanocomposites

Clay-based polymer nanocomposites are often referred to as polymer layered silicates, nanostructured polymers, or simply polymer nanocomposites. These polymers are reinforced with inorganic particles containing at least one dimension in the nanometric scale (<100 nm). Compared to traditional composites (macro- or microscale), polymer nanocomposites offer the opportunity to explore new behaviors and functionalities beyond conventional polymers. Nanoparticles often strongly influence the mechanical properties of polymers in very low volume fractions due to the relatively short distance between nanoparticles, molecular compatibility, and interfacial interaction between the particles and the polymer chains.

**Figure 1:**
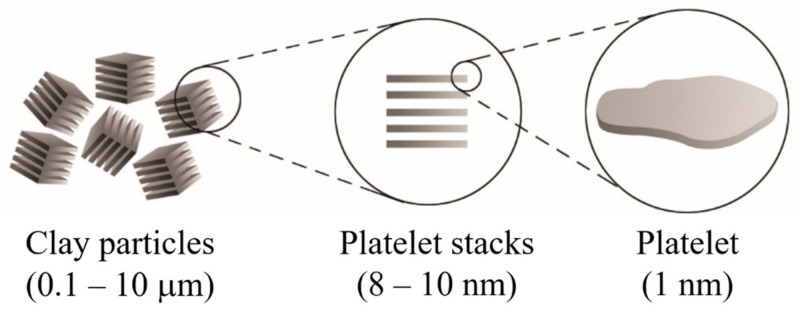
Schematic representation of the arrangement of the platelet stacks that conform to the clay particles.

Clay minerals are known as phyllosilicates, or lamellar silicates, which are the inorganic particles most commonly used to prepare clay-based polymer nanocomposites [2,3,4]. It is necessary to highlight that the clay particles are not by themselves nanometric scale particles, but instead that they are formed by the stacking of several layers that leads to the development of irregular aggregates, as schematized in Figure 1. Each layer has a high aspect ratio between 100 and 800 nm in length and approximately 1 nm in thickness. Its uniform dispersion within the polymer matrix favors developing a very high interfacial area per unit volume, which is the primary reinforcement mechanism of clay-based polymer nanocomposites. However, the layer dispersion mechanism is complex since different aspects must be considered, and that is why the specialized literature focuses on evaluating processing conditions to achieve the maximum level of dispersion [2,5,6,7,8].

Clay layers have a molecular structure based on the stacking sequence. Interesting and complete information is available on the web page of Professor Dorronsoro Fernández from the University of Granada, Spain (Department of Soil Science and Agricultural Chemistry, https://www.edafologia.net/, last visited 12 May 2021).

**Figure 2:**
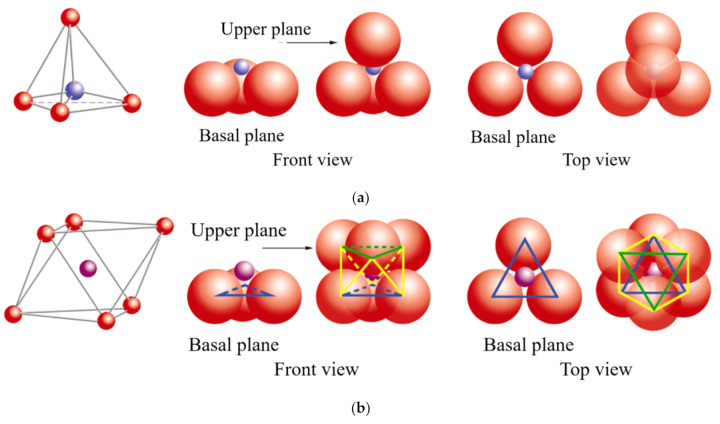
Schematic representation: (**a**) silicon tetrahedron, (**b**) magnesium and/or aluminum octahedron. In both cases, a general diagram is presented, a front view and a top view. 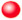 oxygen, 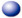 silicon, 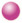 magnesium/aluminum. Adapted from https://www.edafologia.net/, last visited 12 May 2021.

**Figure 3:**
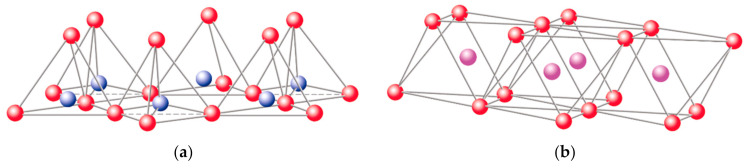
Schematic representation of the structural layers of clays: (**a**) silicon tetrahedron, (**b**) magnesium and/or aluminum octahedron. 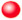 oxygen, 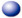 silicon, 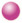 magnesium/aluminum. Adapted from https://www.edafologia.net/, last visited 12 May 2021.

**Figure 4:**
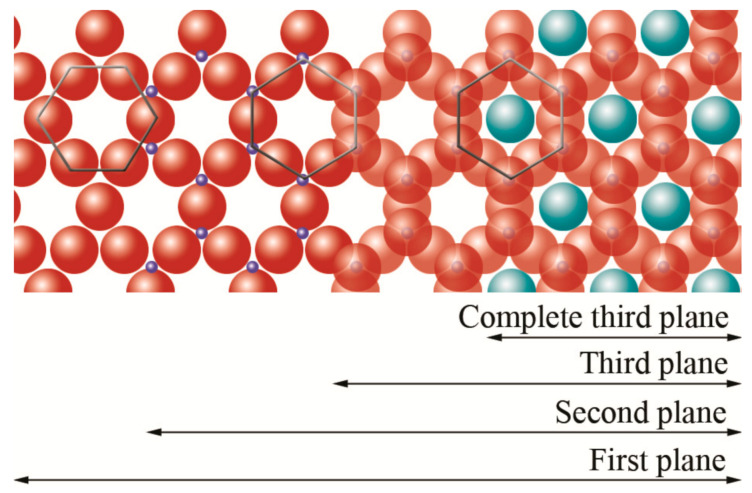
Schematic representation from the top view of the planes formed by the union of tetrahedral and octahedral layers. 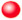 oxygen, 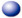 silicon, 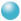 hydroxyl groups. Adapted from https://www.edafologia.net/, last visited 12 May 2021.

The basic compositional unit is the silicon–oxygen (Si-O) tetrahedron, as outlined in Figure 2a. It consists of one silicon cation (Si^−4^) surrounded by four oxygen anions (O^−2^). Chemically, the Si-O tetrahedron has a net electrical charge of −4, (SiO_4_)^−4^, so it is balanced by adding other cations to neutralize their charges (Figure 3a). For this, each vertex of the basal plane belongs to two tetrahedra, since each oxygen is in coordination with two silicones, forming tetrahedral layers distributed under the configuration of hexagons, as can be seen in detail in Figure 4. Sheet silicates (also called layered silicates or phyllosilicates) are obtained when three oxygens on each tetrahedron link to other tetrahedra to form tetrahedral planes.

Sheet silicates are planar structures containing different kinds of layer that can accommodate cations of all sizes. Tetrahedral layers (labeled T in this work) consist primarily of SiO_4_ tetrahedra. Octahedral layers (labeled O in this work) contain divalent and trivalent cations (Mg^2+^ or Al^3+^) in 6-fold coordination, where each octahedron is supported on one of its faces, which represents the octahedral basal plane, as schematized in Figure 2b. Octahedral sheets are composed of individual octahedrons that share edges composed of oxygen and hydroxyl anion groups, with Mg or Al typically serving as the coordinating cation, as presented in Figure 3b. In two dimensions, anions can fit together in symmetrical patterns to form hexagonal patterns (Figure 4).

**Figure 5:**
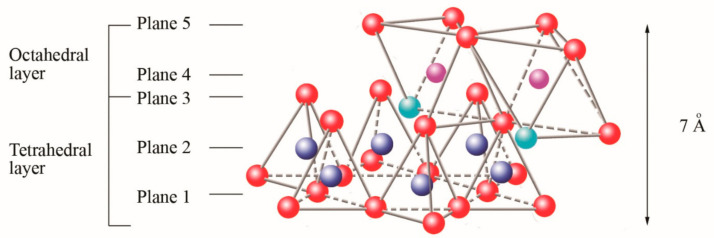
Schematic representation of the final structure corresponding to sheet 1:1. 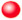 oxygen, 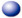 silicon, 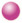 magnesium/aluminum, 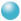 hydroxyl groups. Adapted from [9].

In three dimensions, tetrahedral (T) and octahedral (O) layers may stack in various ways. The arrangement of both layers can be better understood if they are represented through atomic planes, as outlined in Figure 5.

The first plane corresponds to the basal plane of the tetrahedral layer. Silicon atoms are placed in the second plane, occupying part of the space in the basal plane of each tetrahedron (Figure 4). In a third plane, the unshared oxygens (also called apical oxygen) are located just above the silicon, ending up occupying the remaining space, as depicted in Figure 4. In this way, the arrangement of these three planes constitutes the fundamental unit of the tetrahedral layers (Figure 5).

The union between tetrahedral and octahedral sheets occurs with the apical oxygen, linked to Mg^2+^ or octahedral Al^3+^. However, not all the vertices of the octahedral basal plane, formed in part by the apical oxygen, would be shared with the silicon atoms contained in the tetrahedra, so the charge balance occurs when they bind to a hydrogen atom (H), forming hydroxyl groups (OH), as shown in Figure 4 and Figure 5. Thus, the basal plane of the octahedron forms part of the superior plane of the tetrahedra and completes the third plane. It should be noted that all planes represent a hexagonal lattice, while the third plane forms a centered hexagonal lattice, as shown in Figure 4.

**Figure 6:**
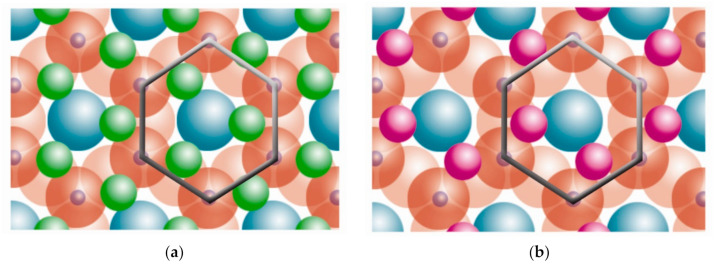
Schematic representation from the top view of the planes: (**a**) trioctahedral, (**b**) dioctahedral. 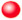 oxygen, 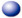 silicon,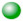 magnesium, 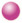 aluminum, 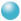 hydroxyl groups. Adapted from https://www.edafologia.net/, last visited 12 May 2021.

The fourth plane consists of the arrangement of octahedral Mg^2+^ or Al^3+^ atoms. These atoms are located in the small free spaces left by every two apical oxygens and one OH group, as shown in Figure 6.

The octahedral Mg^2+^ covers all positions in the trioctahedral plane (Figure 6a). However, the octahedral Al^3+^ covers just two positions of three vacancies, and it is called the dioctahedral plane (Figure 6b). Nonetheless, this plane is within hexagonal networks.

**Figure 7:**
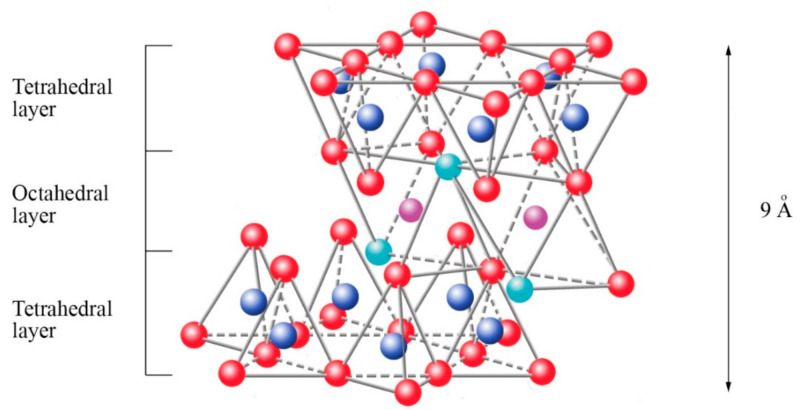
Schematic representation of the final structure corresponding to sheet 2:1. 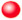 oxygen, 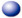 silicon, 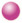 magnesium/aluminum, 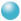 hydroxyl groups. Adapted from https://www.edafologia.net/, last visited 12 May 2021.

The fifth plane corresponds to the superior plane of the octahedra (showed in Figure 5). If the structure ends in this plane, the clay has a T:O sequence (also known as 1:1 structure). However, if another tetrahedral layer is added, a sandwich-type T:O:T sequence is formed. The 1:1 sheet silicate is 7 Å thick, while the 2:1 sheet silicate is about 9 Å thick (Figure 7). Thus, the sheet silicates originate from the stacking of parallel planes with hexagonal symmetries, alternating the planes of ions (O and OH) and cations (Si^4+^, Al^3+^, and Mg^2+^).

**Figure 8:**
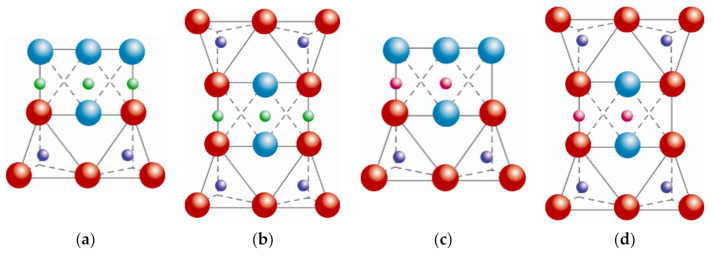
Schematic representation of the arrangement of the atomic planes: (**a**) 1:1 trioctahedral sheets, (**b**) 2:1 trioctahedral sheets, (**c**) 1:1 dioctahedral sheets, (**d**) 2:1 dioctahedral sheets. 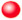 oxygen, 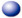 silicon, 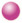 magnesium/aluminum, 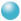 hydroxyl groups. Adapted from https://www.edafologia.net/, last visited 12 May 2021.

Most sheet silicates are monoclinic or triclinic, and have several different polymorphs related to how T:O:T and T:O sheets stack to each other. Figure 8 shows a representative diagram of the 1:1 and 2:1 sheet silicates to better visualize the structural arrangement described. Table 1 shows the classification of clay minerals according to their structural configuration.

Montmorillonite (discovered by Damour and Salvetat in Montmorillon, France) is currently the most widely used mineral clay to prepare polymer nanocomposites. It is a smectite-type clay belonging to the 2:1 sheet silicates and is composed of aluminosilicates (Al^3+^). Montmorillonite clay has a high reaction capacity, exceptional resistance, and a large aspect ratio.

### 2.1. Cation Exchange Capacity

**Figure 9:**
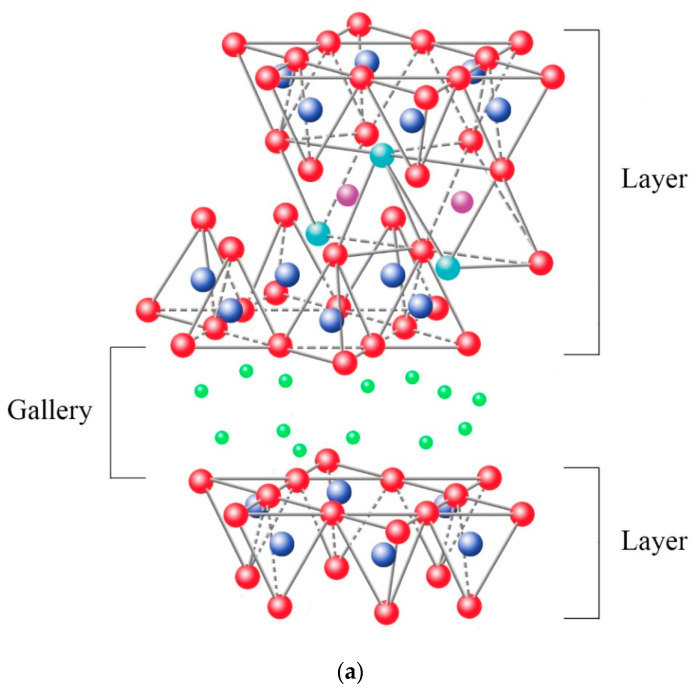

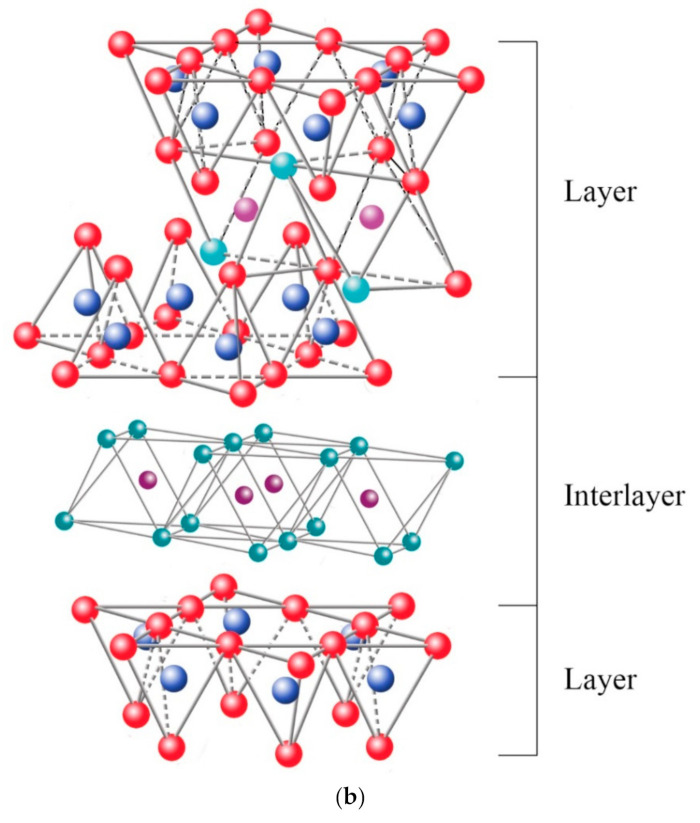
Schematic representation of the clay structure whose charge balance is maintained by: (**a**) interchange cations, (**b**) interlaminar octahedral layer. 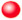 oxygen, 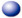 silicon, 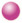 magnesium/aluminum, 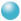 hydroxyl groups. Schematic sketch based on Beyer [136] with permission from Elsevier.

The ability to absorb a certain amount of cations and retain them in an exchangeable state is known as the cation exchange capacity (CEC), expressed in terms of milliequivalents per 100 g (meq/100 g) {Formatting Citation}. The charge of the layer is not locally constant as it varies from layer to layer and must instead be considered an average value over the whole crystal. The importance of knowing the CEC is that the sheets are not electrically neutral due to isomorphic substitutions, where others replace cations such as Si^4+^ with a lower charge (Al^3+^), promoting an excess of negative charge. In this case, the load balance is maintained by the presence of individual cations (as in the micas group) or hydrated cations (as in the case of vermiculites and smectites) in the interlaminar space, which is the existing space between two consecutive sheets, also known as “*galleries*” (Figure 9a). When the hydrated cations are ion-exchanged with organic cations such as more bulky alkylammonium, it usually results in a larger interlayer spacing.

Among the most frequent interlaminar cations are alkalines (Na^+^ and K^+^) and alkaline earth (Mg^2+^). The hydrated cations such as water and different polar liquids increase the interlaminar space by swelling effect. If the interlaminar cations coordinate with OH groups, an octahedral layer would be formed within the interlaminar space (as chlorites, Table 1), developing structures T:O:T:o or 2:1:1, as represented in Figure 9b. In this case, the number 2 represents the two tetrahedral layers, while 1:1 indicates that the layers of the octahedra differ from each other since the interlaminar octahedra do not share vertices with the tetrahedra.

The bonding forces that join the sheets with the interlayer are weaker than those existing between the ions of the same sheet, so the phyllosilicates have a clear parallel direction of exfoliation.

### 2.2. Coupling Agents

In the first instance, clay minerals can only be miscible with hydrophilic polymers. Therefore, the use of coupling agents is necessary to make both phases compatible. These agents are fundamental molecules constituted by a hydrophilic functionality (related to clays) and by an organophilic functionality (related to the polymer), which favors the molecular compatibility between the sheets of the clay and the polymer chains.

The first coupled agents used to obtain nanocomposites were amino acids [137]. However, the most popular are alkylammonium ions, since they can easily be exchanged with the cations in the galleries. The alkylammonium ions are primary alkylamines [138,139]. Its basic formula is:

(1) $$CH_{3}-{\left(CH_{2}\right)}_{n}-NH^{3+}$$

where *n* represents the chain length, which ranges from 1 to 18 carbons. Lan et al. [140] highlighted that the exfoliation of the sheets is favored when ions with a chain length greater than eight carbon atoms are used, while with shorter chains, it led to the formation of agglomerated structures.

**Figure 10:**
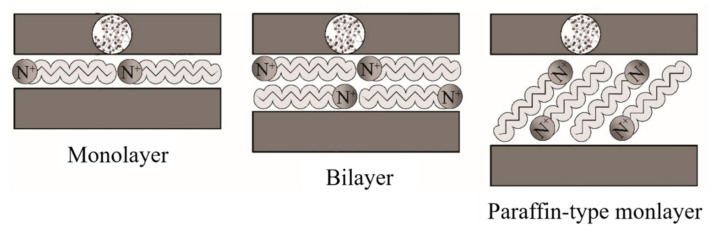
Schematic representation of the configuration of alkylammonium ions within the galleries of clays. Schematic figure based on Lagaly [142] with permission from Elsevier.

Ideally, the alkylammonium ions can be accommodated in various ways within the galleries, depending on the charge density of the clay minerals. Thus, the ions adopt monolayer, bilayer, or paraffin-like monolayers [141], as outlined in Figure 10.

### 2.3. Nanocomposite Structures

**Figure 11:**
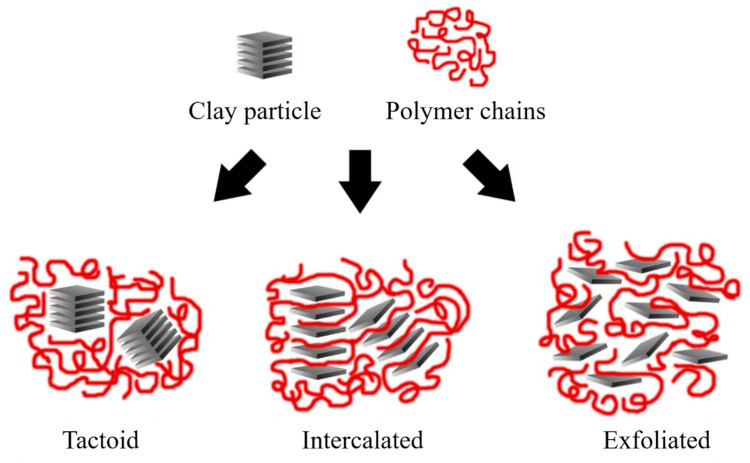
Schematic representation of the nanocomposite structures. Schematic figure based on Beyer [136] with permission from Elsevier.

Depending on the nature of the composite constituents (layered silicate, organic cation, and polymer matrix) and the method of preparation, three main types of composite may be obtained. Thus, polymer nanocomposites can be classified according to their morphology into agglomerates, intercalated, and exfoliated structures, as represented in Figure 11.

Agglomerated composites are formed when the polymer is unable to intercalate between the sheets of clay. In this way, two separate and well-defined phases are obtained, where the sheets remain joined and aligned parallel to each other. It is common to classify the properties of these materials within the microcomposites. Some authors refer to the agglomerated composites as tactoids [2,141] or low-packing nanocomposites since there are no variations in the interlaminar space [2,6].

In the case of intercalated composites (also classified as flocculated [3]), the polymer chains are intercalated between the sheets of the clay, increasing the interlaminar space, obtaining a morphology of multiple highly ordered sheets.

Exfoliated nanocomposites contain sheets entirely separated and dispersed within the polymer matrix. This type of exfoliated structure presents relevant mechanical properties due to the high aspect ratio of single sheets. However, this structure is difficult to obtain, and three main processing strategies are commonly used.

### 2.4. Polymer Intercalation in Solution

**Figure 12:**
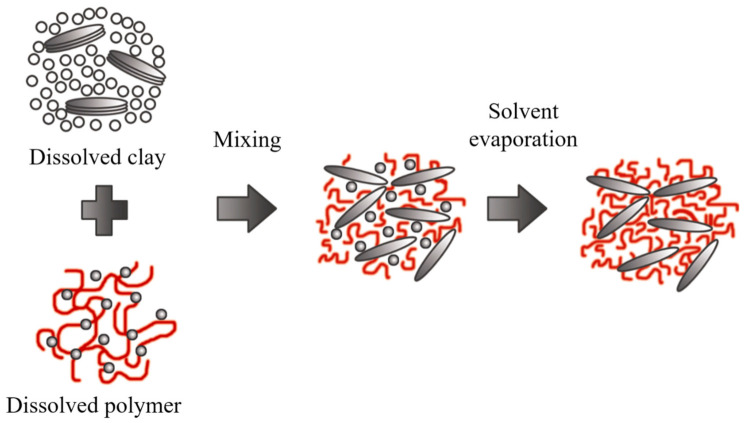
Schematic representation of polymer intercalation in solution. Schematic figure based on Zanetti [143] with permission from John Wiley and Sons, and Unalan [144] from RSC Adv. Open Access.

The clay mineral is suspended in a polar organic solvent such as water, toluene, ethanol, etc., forming a gel-like structure. Subsequently, the polymer is dissolved and dispersed in the same type of solution, and the reaction is initiated by mixing the solutions, where polymer chains start to fill spaces in the galleries. Then, the solvent is removed by evaporation, obtaining the nanocomposite with a multilayered structure, as outlined in Figure 12.

The nanocomposites obtained through this method are highly selective since the polymer and clay minerals possess different physical and chemical properties. The solvent is also essential because it is expensive and not environmentally friendly for large-scale production.

### 2.5. In Situ Polymerization

**Figure 13:**
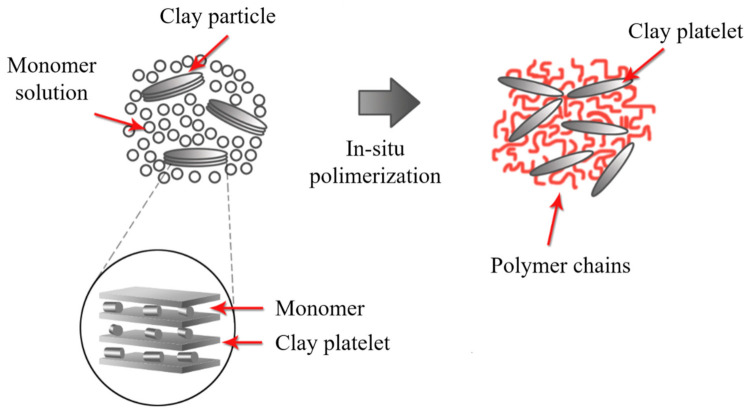
Schematic representation of in situ polymerization. Schematic figure based on Zanetti [143] with permission from John Wiley and Sons, and Unalan [144] from RSC Adv. Open Access.

In this method, the galleries are expanded using a liquid monomer or a monomer in solution. The polymerization starts by the diffusion of an organic initiator or by catalysis through exchangeable cations (Figure 13) [145]. After polymerization termination, the solvent is evaporated, and the nanocomposite is ready for further modification. The in situ polymerization should be the best method to obtain high intermolecular distance between the clay layers. However, this method contains similar drawbacks to the solution-based method due to large-scale difficulties and environmental considerations.

### 2.6. Melt Blending Process

This method consists of mixing both phases (polymer and clay minerals) under the action of a high-temperature shear force. Melt intercalation is used for synthesizing thermoplastic polymer nanocomposites at a large scale. This procedure is compatible with industrial processes such as extrusion, making it more economical, convenient, and environmentally friendly because solvents are not required. However, the high temperatures during the extrusion process (up to 220 °C) can degrade the coupling agents (clay minerals modified with alkylammonium).

In a broad definition, the extrusion process refers to any transformation operation in which molten material is forced through a die to produce an article of constant cross-section and, in principle, indefinite length. In addition to plastics, many other materials are processed by extrusion, such as metals, ceramics, or food, obtaining very varied products such as aluminum or PVC window frames, pipes, pasta, etc. From a plastics point of view, extrusion is one of the essential transformation processes. The polymer is generally fed in solid form (commonly dust or pellet) in the hopper section and exits the extruder in the molten state. On some occasions, the polymer can be fed in molten form from a reactor where the extruder acts as a pump, providing the necessary pressure to pass the polymer through the nozzle. The extrusion process is frequently used to mix distinct materials, additives, and fillers to add better performance, reduce costs, and obtain multiple functionalities. These new formulations are further processed to create components or preforms using injection molding, blow molding, or thermoforming techniques.

Although there are various types of extruders, the most widely used are single-screw and twin-screw extruders. Specifically, a single-screw extruder can perform six main functions: transporting the solid material towards the melting zone, melting the polymer, pumping the melt, mixing, degassing, and forming. However, not all of the above functions necessarily take place during the operation of the extruder. According to the purpose, the extrusion process starts with the material feeding system, a melting-plasticizing system, the pumping, and a pressurization system, generating a mixing effect.

It is common to find in the literature that single-screw extruders have poor material mixing due to their design. However, it is essential to consider many other factors that affect the end product, such as the wear of extruder working parts, rotational speed, pressure, nozzle type, and many more. In addition, the extruders are not just used for mixing but also for producing various materials, e.g., direct molding at the nozzle, injection into the die, etc. Ekielski et al. [146] evaluated the wear status of the single-screw extruder working elements based on die pressure and screw load values changes. The changes to these parameters were analyzed as a frequency spectrum using wavelet analysis tools. Due to the dynamic characteristics of the process in determining natural frequencies, the authors used the Morlet wavelet transform, observing that it is possible to accurately evaluate the degree of wear of the friction elements in a single-screw extruder.

Extrusion technology is also used in the food processing industry, known as extrusion-cooking, to produce so-called engineered foods and special feed. Leszek Moscicki and Dick J. van Zuilichem detailed an interesting work related to extrusion-cooking using single-screw extrusion technology [147]. The authors mentioned that the shear exerted by the rotating screw and the additional heating of the barrel promote a rheological modification. The physical aspects such as heat transfer, mass transfer, impulse transfer, residence time, and residence time distribution have a substantial impact on the properties of food and feed during extrusion-cooking and can drastically influence the quality of the final product.

**Figure 14:**
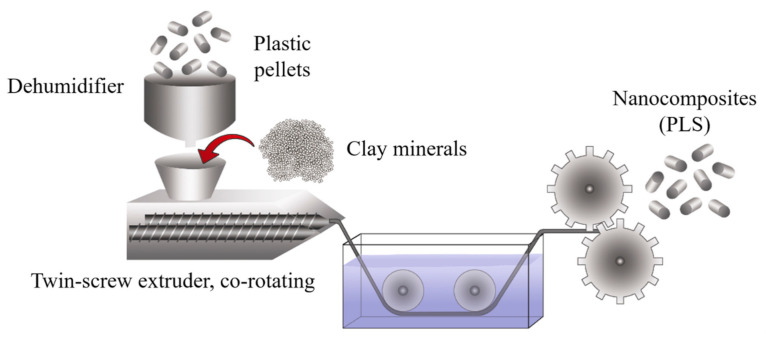
Schematic representation of the production process using twin-screw extrusion.

Twin-screw extruders provide a much higher degree of shear than single-screw extruders, and the screw rotation can be co-rotating or counter-rotating. Therefore, this process can be too aggressive for some applications; even so, the high shear promotes twin-screw extrusion to prepare clay-based polymer nanocomposites (Figure 14). However, the single-screw extrusion should be considered for producing starch-based bionanocomposites or other natural composites.

**Figure 15:**
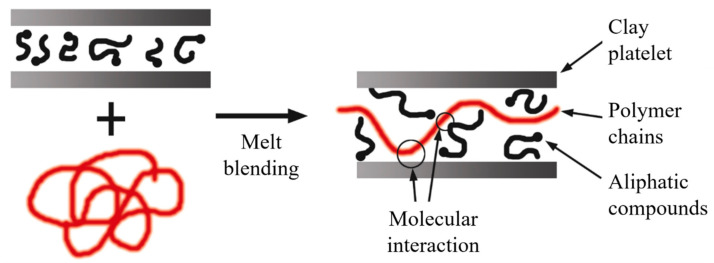
Schematic representation of intercalated structures for melt blending. Schematic figure based on Vaia [148] with permission from American Chemical Society, Copyright 1997.

The literature is consistent in stating that the extrusion process leads to the development of intercalated structures. However, exfoliated structures can develop if there is high molecular compatibility between the phases, and in many instances, the use of additives is required (Figure 15).

Some authors [149,150,151] evaluated the influence of the extrusion process on the morphology of polymer nanocomposites. In this way, Dennis et al. [150] observed that a single screw extruder did not provide sufficient shear to separate or fracture the clay layers and did not offer an adequate residence time for layer dispersion. On the other hand, intercalated structures and some agglomerations are present when using a twin-screw extruder under the co-rotating configuration. By using the counter-rotating configuration, a high level of exfoliation was achieved.

Fornes et al. [151] found that the design of the extrusion screws also conditioned the morphology of the nanocomposites. The low- and medium-cut spindles developed interleaved structures, while a high-shear design obtained a high level of exfoliation.

Based on the previous studies, it is possible to consider that using a counter-rotating twin-screw extruder with high- or medium-shear screws should favor the exfoliation of the clay sheets. Nonetheless, it is important to consider that factors such as molecular compatibility and good processing conditions (dosage rate, temperature, and residence time, among others) are necessary for optimal exfoliation [152].

**Figure 16:**
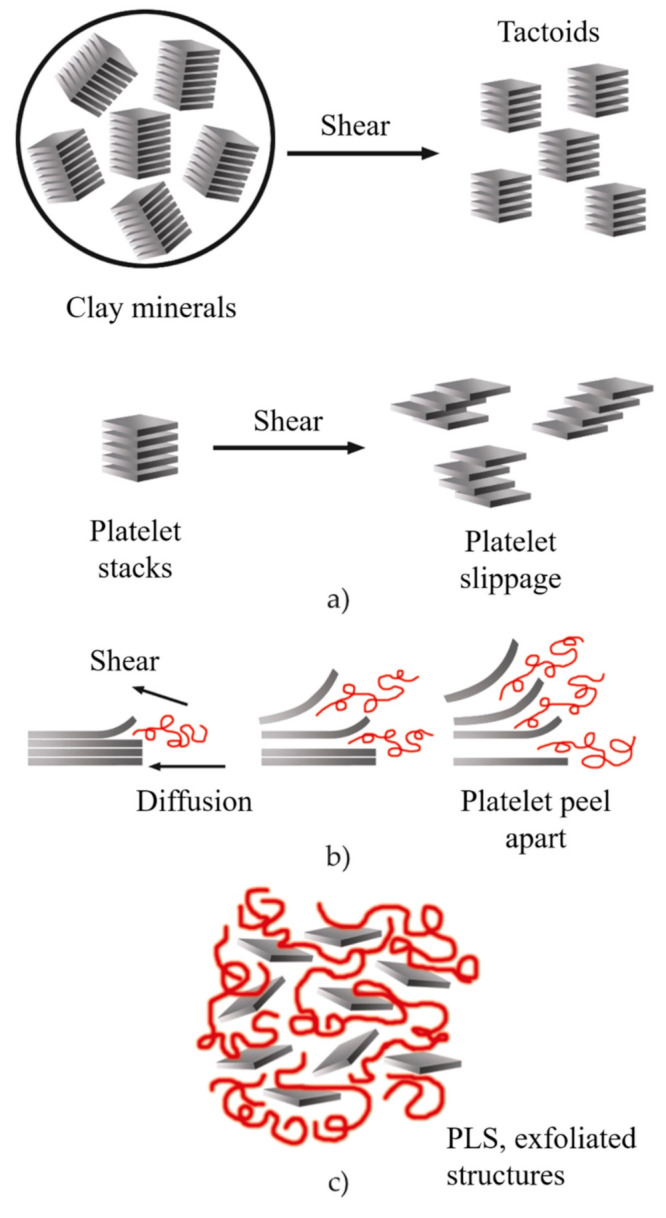
Schematic representation of the exfoliation mechanism of the clay platelets during the twin-screw extrusion process: (**a**) fracture and sliding of the sheets, (**b**) intercalation, (**c**) exfoliation. Schematic figure based on Fornes [154] with permission from Elsevier.

During the twin-screw extrusion process, the exfoliation mechanism begins with the fracture of the particles and the sliding of the sheets until they become stacked sheets of smaller size, as schematized in Figure 16a. This first phase requires a high shear intensity. Subsequently, the polymer is sandwiched between the sheets, taking advantage of their flexibility to increase the distance between them. This second phase requires both high shear and good molecular compatibility (Figure 16b). Finally, the exfoliated sheets are randomly dispersed within the matrix (Figure 16c), requiring adequate residence time [8,153].

It is important to note that an intense shear does not guarantee a more significant number of exfoliated sheets. Similarly, a longer residence time does not provide better dispersion. For this reason, a large number of studies have focused on developing processing conditions that allow for increasing the level of exfoliation by using the melt intercalation process [1,5,8,151,153,155].

**Figure 17:**
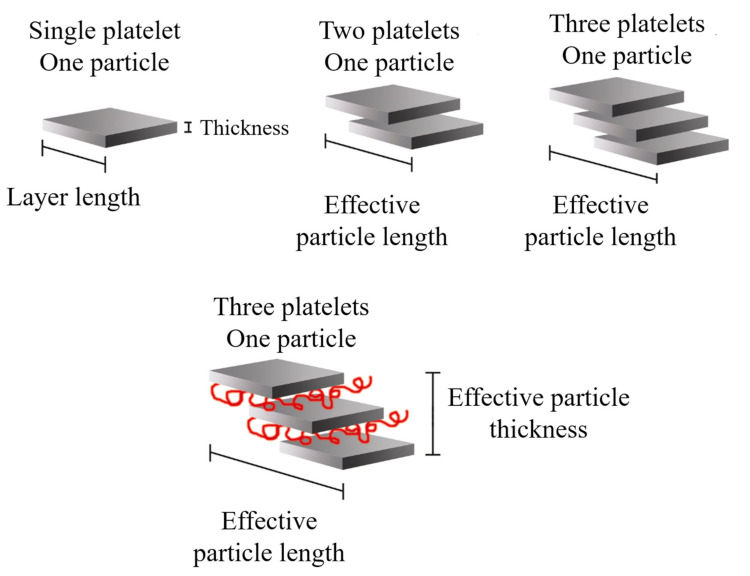
Schematic representation of the effective length and thickness of the clay particles present in nanocomposites with a certain degree of intercalation. Schematic figure based on Fornes [154] and Chavarria [156] with permission from Elsevier.

It is possible to consider the average dimensions of each particle: length (*ℓ*_p_), thickness (t_p_), and aspect ratio (*ℓ*_p_/t_p_). Some authors [8] detailed that the increase in *ℓ*_p_ can be related to the sliding of the sheets that occurred during the twin-screw extrusion process, which can be defined as effective particle length, as shown in Figure 17.

The clay-based polymer nanocomposites have gained the attention of academics and industry in recent decades. Integrating small percentages of clay minerals into the polymer matrix improves the mechanical properties compared to neat polymers. Properly dispersed and aligned clay platelets have proven to be very effective for increasing stiffness without altering the polymer density. There is extensive literature regarding the mechanical properties of polymers enhanced by low clay content, as summarized in Table 2.

## 3. Fracture Mechanics

Fracture mechanics is based on the need to analyze the flaws present due to a part or component’s manufacturing and machining processes. Different manufacturing processes induce failures or small internal or surface cracks, and the fracture mechanics allows for characterizing those cracks that can propagate (instability) and cause failures in the structure.

**Figure 18:**
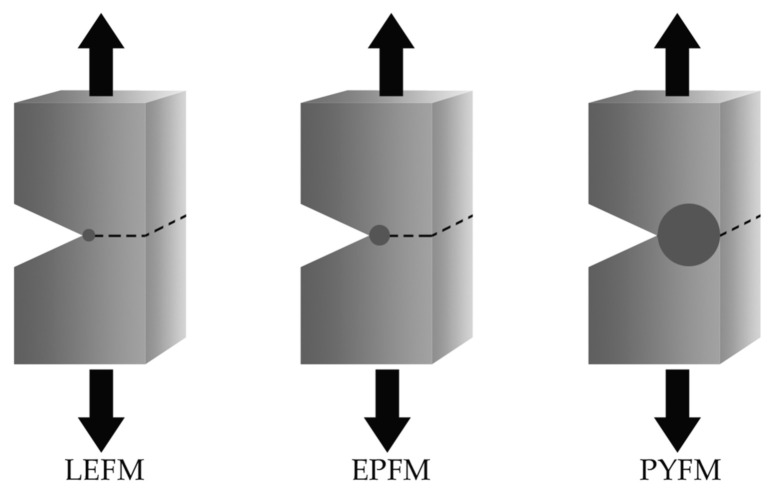
Schematic representation of the three approaches to fracture mechanics: linear-elastic fracture mechanics (LEFM), elasto-plastic fracture mechanics (EPFM) and post-yield fracture mechanics (PYFM). The shaded area represents the extent of the plastic deformation that develops during the fracture process.

Fracture mechanics involves three complementary theories: linear elastic, elastic-plastic, and post-yielding fracture mechanics. Practically, the main difference is in the development of plastic deformations around the crack-tip of notched specimens during the fracture process, as presented in Figure 18.

**Figure 19:**
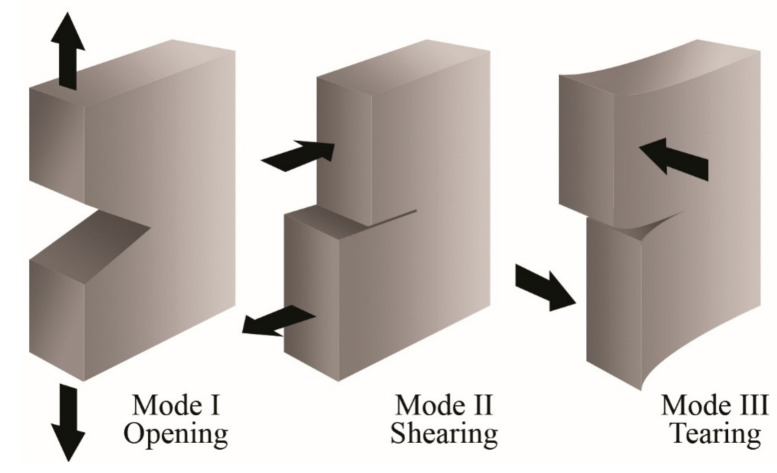
Schematic representation of the three modes for crack propagation.

Generally, a crack in a solid body can propagate in three different ways depending on the applied load and with respect to the crack plane, as shown in Figure 19. Mode I (opening mode) represents the opening of the crack due to stresses perpendicular to the plane of the crack. Mode II (sliding mode) refers to the displacement of the crack under shear forces that act parallel to the plane of the crack and perpendicular to the crack front. Mode III (tear mode) is produced by shear forces that act perpendicular to the plane of the crack and parallel to the crack front. It is common to indicate the fracture mode employing a subscript I, II, or III.

Fracture mechanics theory is relatively new, beginning in 1913 when Charles E. Inglis [194] studied the stress behavior of plates with ellipse-shaped defects. Considering an infinite isotropic plate in uniaxial tension, Inglis proposed the linear elastic solution for the ellipse’s tension field. Later, in 1920, Griffith [195] used the work of Inglis to calculate the stress concentrations around the ellipse and to predict the resistance to fracture. However, Inglis’s solution poses a mathematical difficulty considering that the stresses approach infinity at the crack-tip in the limit of a perfectly sharp crack, which is physically impossible. Thus, Griffith proposed the energy-balance approach, which is based on balancing the reduction in potential energy during fracture with the increase in surface energy due to creating new free surfaces when a crack grows. Griffith’s theory provides excellent agreement with experimental data for brittle materials and is the fundamental base for the linear elastic fracture mechanics (LEFM) theory.

After Griffith’s work, in 1950, G. R. Irwin observed that ductile materials develop a plastic zone at the crack-tip. The plastic zone increases with the applied load and leads to the dissipation of energy in the form of heat. Therefore, a dissipative term must be added to the energy-balance relationship for brittle materials. In physical terms, ductile materials require additional energy for crack growth. Thus, Irwin extended the theory to some ductile materials through the concept of energy release rate (Gc) [196]. Subsequently, Irwin used Westergaard’s approach [197] to demonstrate that local stresses and displacements near the crack-tip had a general solution. He introduced the stress intensity factor (K), which relates the local mechanical state near to the crack-tip with macroscopic characteristics in a continuous process of stable crack growth until the onset of instability.

After the fundamentals of linear elastic fracture mechanics (LEFM) were established (around the 1960s), several scientists began to study the plasticity that developed at the crack-tip. Wells [198], in 1961, presented the concept of crack tip opening displacement (CTOD) for materials that can show some plastic deformation at the crack-tip during the fracture process. In 1968, Rice [199] developed the J-integral approach as a parameter for the characterization of the crack propagation under elastic-plastic fracture mechanics (EPFM) conditions. The J-integral initially emerged as a fracture criterion for small-scale plasticity conditions at the crack-tip. Later, it was considered a fracture criterion in large-scale plasticity for fracture initiation and stable crack growth. The J-integral approach considers the elastic and plastic parts separately to characterize near-crack-tip deformation filed in linear and non-linear elastic materials. Experimentally, the J-integral shows an energetic contour path integral, independent of the path around a crack, and it enables determining the strain energy release rate. The J-integral and CTOD are the parameters most often used to correlate mechanically short and long crack growth rates.

Broberg [200,201], in 1968, observed changes in the distribution of plastic deformation near the crack-tip in materials with considerable plastic deformation before fracture. These changes contribute to a gradually increasing screening of the energy flow through the plastic region towards the crack-tip, leading to stable crack growth accompanied by plastic collapse. Thus, Broberg established the beginning of the post-yielding fracture mechanics (PYFM) criteria based on the stable crack growth.

In 1977, Cotterell and Reddel [202] developed Broberg’s ideas and proposed the essential work of fracture (EWF) method, which allows for determining the fracture properties of ductile materials.

### 3.1. Essential Work of Fracture

The EWF approach considers the total energy involved during the ductile fracture process, where the plastic deformation is fully developed around the ligament region before the crack growth. The EWF consists of using deeply double notched in tension (DDENT) specimens to determine the energy absorbed by fracturing the DDENT specimens. The total fracture energy (*W_f_*) can be separated into two terms: the essential work of fracture (*W_e_*) performed in the inner fracture process zone (FPZ) and the non-essential work of fracture (*W_p_*) performed in the outer plastic zone (OPZ):

(2) $$W_{f}=W_{e}+W_{p}$$

**Figure 20:**
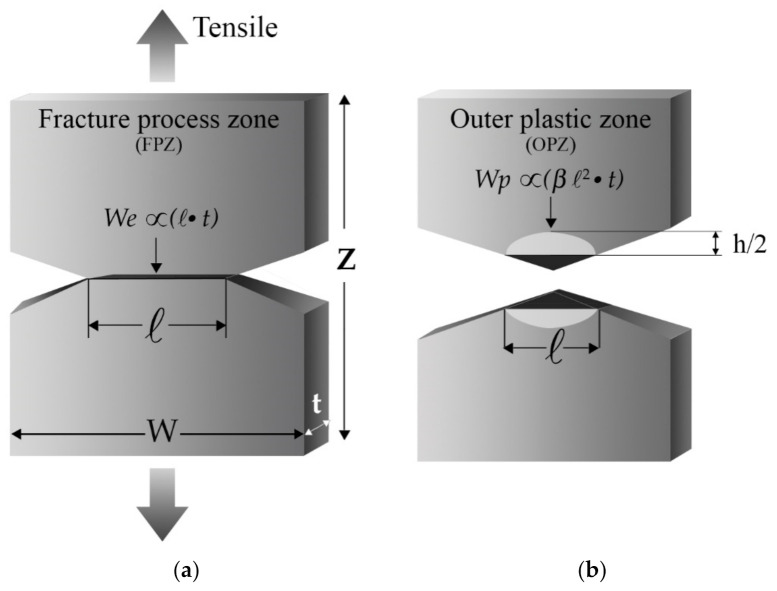
Schematic representation of the DDENT specimen: (**a**) before being tensile tested and (**b**) after being tensile tested.

Both areas are easily identifiable, as outlined in Figure 20.

*W_e_* is associated with the FPZ (where the real fracture process occurs) and represents the energy involved in creating two new surfaces during the crack propagation. This term is proportional to the ligament section *ℓ* · *t*, as depicted in Figure 20a.

The *W_p_* term is also named “plastic work” and is related to the OPZ, where the rest of the phenomena associated with the ductile fracture, such as plastic deformation, and other dissipative processes such as shear, crazing, or cracks, occur. This term is proportional to the volume of the deformed region (Figure 20b).

The previous concepts enable rewriting the Equation (2) in the following expression:

(3) $$W_{f}=W_{e}+W_{p}=w_{e}\cdot \ell \;t+\beta \cdot w_{p}\cdot \ell^{2}\;\cdot t$$

where *w_e_* is the specific essential work of fracture per unit area of the ligament (*ℓ*·*t*), *ℓ* is the length of the ligament, *t* is the thickness of the specimen, *β* is the shape factor of the plastic zone, and *w_p_* is the specific non-essential work of fracture per unit volume (*ℓ*^2^·*t*). Dividing both terms of Equation (3) by the ligament section (*ℓ*·*t*), we find that the specific work of fracture work (*w_f_*) is:

(4) $$w_{f}=\frac{W_{f}}{\ell \;t}=w_{e}+\beta w_{p}$$

**Figure 21:**
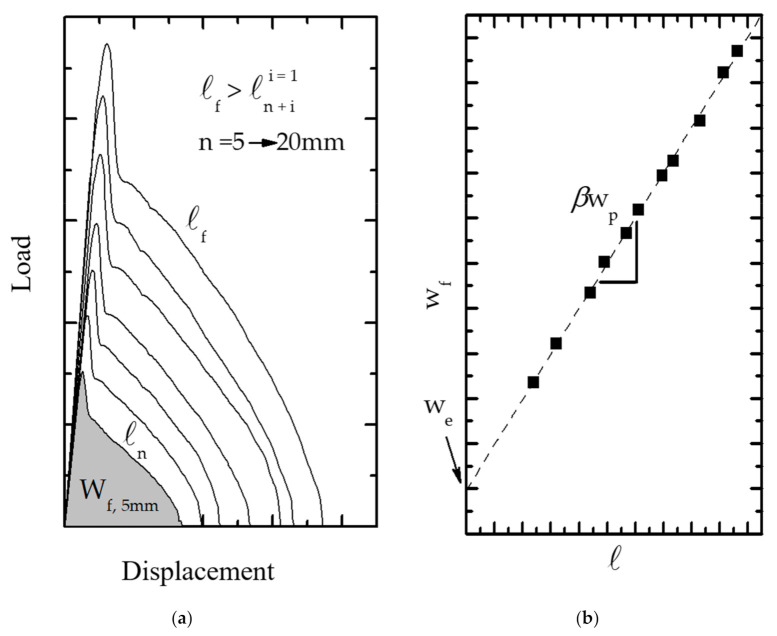
Schematic representation of the EWF fracture process: (**a**) the shape of the L–d curves, necessary to represent (**b**) wf vs. *ℓ* and obtain the linear regression.

The experimental procedure consists of testing several DDENT specimens with different ligament lengths. The fracture energy (*W_f_*) is determined as the area under the experimental load vs. displacement curves (Figure 21a).

According to Equation (4), we can calculate *w_e_* and *βw_p_* terms by representing the *w_f_* values in front of *ℓ*. These points are subject to linear regression, where *w_e_* is the intercept of the fitting line with the y-axis, while *βw_p_* is the slope of the fitting line, as presented in Figure 21b.

Theoretically, *w_e_* is an intrinsic material constant that depends only on the thickness and is equivalent to J_IC_ [203], which has been supported experimentally by different authors [204,205,206,207] and contrasted with the CTOD values [17]. However, compared to the J-integral approach, the EWF method has certain procedural advantages, such as its simplicity in experimental testing or its applicability to very thin thicknesses such as thin sheets or films [208,209].

The EWF method is currently attractive for several research projects that deal with fracture toughness for polymer materials. However, its application is limited since there are currently no mathematical models that allow the use of the data obtained by the EWF technique to simulate the fracture of ductile materials. Therefore, it is necessary to highlight the works performed by Knockaert, Pardoen, Chen, and Cotterell [207,210,211,212] in the numerical simulation for the fracture of DDENT-type specimens. Furthermore, Chen et al. [213] investigated the correlation between fracture parameters and the molecular structure of amorphous and ductile polymers.

It is also noteworthy that the application of the EWF method has been extended from Mode I to the other two crack propagation modes: shear (Mode II) [214,215] and tear (Mode III) [216,217]. Additionally, the EWF method was applied to evaluate the adhesion energy in joints [218,219].

#### 3.1.1. Experimental Considerations for the EWF Method

In 1993, Gray [220] made the first attempt to standardize the EWF method within the technical committee number 4 of the European Structural Integrity Society (ESIS TC-4), where the following recommendations were established:

- Under tensile load, the full ligament yielding should be reached before the crack is initiated
- Equation (3) can be applied if the DDENT specimen is in a plane stress fracture condition, which is verified by applying Hill’s criterion [221].
- Self-similarity of the experimental load–displacement curves for each *l*, which supports the development of a fracture geometry common to all DDENT specimens.
- For materials with ductile fracture, the FPZ undergoes a necking process which then breaks into a fracture surface. So, the required conditions involve the specimen thickness (*t*), the width of DDENT specimen (*W*), and the plastic zone size (*r_p_*):
  (5) $$\left(3-5\right)t\leq \ell \leq \min\left(\frac{W}{3}\right)or\;2r_{p}\;\;\;\;\;$$
  where *r_p_* is the radius of the plastic zone, which is defined as
  (6) $$2r_{p}=\left(\frac{\pi }{8}\right)\left(\frac{Ew_{e}}{\sigma_{y}^{2}}\right)$$
  where *E* and *σ_y_* are the elastic modulus and the tensile strength, respectively.

In addition, the height of the plastic zone (h in Figure 20) must be measured to determine the shape factor (*β*), where three primary geometric forms were established: rhombus, circle, or ellipse.

This first standard protocol was revised in 1997 and later in 2001 [222], emphasizing that the similar stress condition could improve Hill’s criterion. This is verified through σ_max_, which must be similar in all the tested specimens, regardless of *ℓ*.

Despite all the reviews and work carried out by the TC-4 committee of the ESIS, some issues cause controversy, representing a challenge for research. Table 3 lists some of these topics.

#### 3.1.2. Dimensions of the DDENT Specimen

According to the theory of the essential work of fracture, *w_e_* represents the material’s toughness, and so it is independent of specimen geometry. Maspoch et al. [245] detailed the effect of the DDENT specimen dimensions by applying the EWF method testing an isotactic polypropylene (iPP). They concluded that the width of the specimen does not drastically influence the values of *w_e_* or *βw*_p_, which would indicate that both fracture parameters are intrinsic properties of the material. Similarly, the length of the specimen does not seem to influence the EWF parameters. Regarding *ℓ*, the authors observed that the minimum length does not seem to be subject to the criterion *ℓ* > (3–5)t, since *ℓ* was valid for values between 5 and 6 mm, and independent of the specimen thickness. Finally, the thickness had a significant effect on the fracture parameters of the iPP studied, since an increase in t produced an apparent decrease in the values of *w_e_* that may be related to both the morphology and the fracture mechanisms, as could be appreciated through scanning electron microscopy (SEM).

#### 3.1.3. Use of Video Extensometer

The essential fracture work theory indicates that all the energy involved during the fracture process is absorbed in both the fracture process zone (FPZ) and the plastic zone (OPZ). However, it is possible to argue that part of the viscoelastic energy can be stored in the DDENT specimen (outside the OPZ), and is released slowly after the fracture [246]. Fung et al. [247] demonstrated that the viscoelastic energy is not included in total fracture energy and proposed to measure the plastic deformation using a videoextensometer. They found the use of videoextensometer does not significantly affect the values of *w_e_* but reduces the *βw*_p_ values.

Other authors detected and quantified the plastic deformations during the EWF test using a digital image correlation system (GOM-Aramis™) that allowed visualizing the field of deformations in 3D.

One of the most common interests for experimental fracture mechanics characterization is evaluating the influence of the notching quality on the fracture parameters. Martinez et al. [244] proposed a new procedure for sharpening the notches of the DDENT specimens, which was based on pulsed laser ablation in periods of femtoseconds (femtolaser). This procedure was compared to the conventional notch sharpening method by a razor blade. The results show that the radius of the bottom of the notch appears to be critical below 10 μm. However, the fracture parameters are susceptible to plastic deformation induced during notching, altering the *w_e_* values.

#### 3.1.4. Energy Partitioning

**Figure 22:**
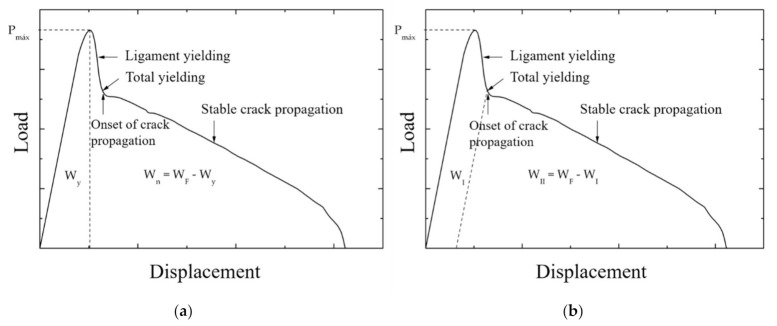
Energy partitioning methods: (**a**) method at yield, (**b**) method of initiation.

According to the EWF theory, *w*_f_ considers all the energy dissipated divided by the ligament section *ℓ*·*t* (Equation (3)), and the fracture process is related to the shape of the load–displacement curves. Therefore, it is possible to suggest the energy contribution separation in terms of fracture initiation and propagation. Mai et al. [248] partitioned the total work into two distinct parts: the energy for initiate fracture and the fracture work for crack growth, including crack propagation and necking. Karger-Kocsis et al. [249] proposed that the maximum load (Figure 22a) divides the fracture energy into two components: the yield work (*W_f,y_*) and the necking and propagation work (*W_f,n_*):

(7) $$w_{f}=w_{f,y}+w_{f,n}=\left(w_{e,y}+\beta w_{p,y}\cdot \ell \right)+\left(w_{e,n}+\beta w_{p,n}\cdot \ell \right)$$

Ferrer-Balas et al. [250] proposed the initiation work method, limiting the partition energy at the end of necking (Figure 22b), just before starting the crack propagation. Thus, the fracture energy is separated into two terms, WI (initiation process, which refers to the full ligament yield, which is a phenomenon of plastic deformation) and WII (crack propagation and energy dissipation in the plastic zone). The elastic energy absorbed during the ligament yield is consumed during the crack propagation; hence, it is considered part of WII (propagation) and is subtracted from WI (initiation), as schematically depicted in Figure 20.

(8) $$w_{f}=w_{f,I}+w_{f,II}=\left(w_{e,I}+\beta w_{p,I}\cdot \ell \right)+\left(w_{e,II}+\beta w_{p,II}\cdot \ell \right)$$

#### 3.1.5. Proposal for the New Shape of the Plastic Zone

**Figure 23:**
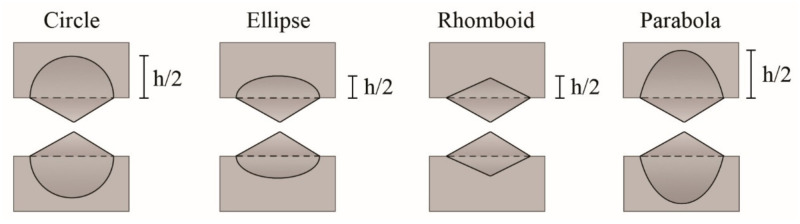
Schematic representation of the different ways in which the plastic zone can develop during the process of fracturing DDENT specimens. Schematic figure based on Ferrer-Balas [251] with permission from Elsevier.

Under similar geometric and test conditions, the plastic term (*βw_p_*) can be used as a comparative parameter between different materials. The shape factor depends on the material and is related to the geometry of the plastic zone developed during the fracture of the DDENT specimens. On the other hand, the value of *w_p_* cannot be determined directly through the linear regression obtained from the graphical representation of *w_f_* vs. *ℓ*, which justifies the need to measure *β* accurately. It is common to observe that the plastic zone develops an intermediate shape between elliptical and rhomboid geometries [251], identified as the intersection between two parabolas and defined using the following equation:

(9) h=k·β·ℓ

where *k* is a constant that depends on the shape of the plastic zone and takes values of 1.27 for circular and elliptical geometries, and 2 and 1.5 for rhomboid and parabolic shapes, respectively (Figure 23).

In this way, *β* can be determined as the slope of the linear regression obtained by representing the total height of the plastic zone (*h*) versus the ligament length (*ℓ*), divided by the factor k.

The EWF method allows us to relate the fracture processes with distinct microstructural parameters. Thus, some studies related to complex phenomena such as aging or degradation [252,253] and the ductile–brittle transition [254,255] use the EWF approach. 

The high sensitivity of the EWF method makes it of particular interest to study the processing–properties relationship to optimize the processing parameters [256], and even to evaluate the influence of annealing in different polymer systems [236,257,258]. Furthermore, the EWF technique is appropriate to study the influence of different morphological parameters such as molecular weight, anisotropy, molecular entanglement density, and crystalline structure have on the fracture toughness of films and thin sheets.

Even though the numerous published works demonstrate the satisfactory application of the EWF on ductile materials (polymers and metals included), there are still some controversies about the influence of some experimental variables such as the test speed, the DDENT specimen thickness, and the notching quality, which may limit the applicability of the EWF method.

### 3.2. EWF for Clay-Based Polymer Nanocomposites

Recently, the EWF method began to be used in clay-based polymer nanocomposites [188,224,259,260,261]. The high sensitivity of the EWF technique is attractive to evaluate the influence of distinct variables such as the type of organic modifier, the filler content, the platelets’ orientation, and the exfoliation degree on the fracture toughness of polymer nanocomposites. Table 4 presents a summary of the literature found for the applicability of the EWF method in polymer nanocomposites.

Polyolefins such as polypropylene and polyethylene are the most common thermoplastics for EWF evaluation. Similarly, montmorillonite is a clay mineral mainly used as a reinforcement for thermoplastic resins.

Saminathan et al. [232,233] evaluated the effect of loading rates on the EWF parameters. The PP/MMT nanocomposites were prepared by melt mixing using a twin-screw extruder. The compatibilizer was maleic anhydride grafted polypropylene (MA-g-PP). The commercial nanoclay Closite^®^15A was used as a nanofiller. The concentration of nanoclay was 5 wt.%, and the ratio between the nanoclay and MA-g-PP was 1:1. The nanocomposites were injection molded to obtain 1.5 mm thick plates. The EWF tests were conducted at room temperature with loading rates varying from 1 to 20 mm/min. The authors mechanically characterized the nanocomposite. The results obtained show an increase in tensile modulus, flexural modulus, yield strength by 25%, 20% and 10%, respectively, compared to pure PP. Nevertheless, the authors observed the maximum percent strain reduced by 41%. According to the EWF results, the authors revealed that the specific work of fracture increases 25% with respect to pure PP, and as the loading rate increases, the specific EWF for yielding increases, but the specific EWF for necking decreases.

Nekhlaoui et al. [259] studied the fracture of polypropylene nanocomposites. In this work, the authors compared the fracture behavior using the EWF approach in PP mixtures with and without a compatibilizer. The nanofiller percentage was 5, 10, 15, 20, 25, 30 and 35 wt.%, through the intercalation method with PP and PP–SEBS–g–MA. The DDENT specimens were obtained by injection molding with a thickness of 2 mm. The authors observed that the presence of clay particles at higher content (30 wt.%) leads to a reduction in both the essential and plastic work of fracture due to the rigid character of the clay.

The EWF approach is widely used to determine the plane stress fracture toughness of highly ductile polymers. To understand how the presence of nanofillers influences fracture toughness, Karger-Kocsis et al. [269] mixed amorphous copolyester and polypropylene block copolymer with multiwall carbon nanotube (MWCNT), graphene (GR), boehmite alumina (BA), and organoclay (oMMT) at one wt.% each. They performed the EWF test, and the data reduction occurred by energy partitioning between yielding and necking. The fracture zone showed some tearing morphology. The EWF prerequisites were not met with the nanocomposites containing MWCNT and GR, by contrast to those with BA and oMMT. Therefore, the fracture toughness of nanocomposites with homogeneous clay dispersion was properly determined using the EWF method. The authors also found that incorporating oMMT may result in an adverse effect between the *w_e_* and *w_p_* terms.

Some authors [265] used the EWF method to compare the effect of the clay content and the coupling agent on the fracture toughness of polypropylene films. The authors found that the addition of mineral clay led to a considerable reduction in the *w_e_* term compared with the unfilled PP, and the coupling agent did not influence the fracture parameters. However, the presence of clay promoted an appreciable increase in dissipated plastic work, regardless of the clay content.

Other authors applied the EWF method to evaluate molecular compatibility, morphology, and clay content on the fracture parameters [178,180,213,232,233,259,260,310,311].

Bureau et al. [263] discussed the failure mechanisms of polypropylene/clay nanocomposites. The fracture behavior of these nanocomposites, based on polypropylene with organo-modified clays (2 wt.%) and different coupling agents, was evaluated using the EWF procedure. The microstructure revealed an acceptable level of intercalation with partial exfoliation (<100 nm). Furthermore, the mechanical properties increased by 25–50% due to the reinforcing effect of the nanoparticles. The authors evaluated the trends observed in the specific work of fracture for the crack initiation–ligament length (*w_e_*_,init_ and *βw_p_*_,init_). The results show a flat slope of the *w_e_*_,init_ curve obtained for PP, which was caused by the difficulty of developing a plane stress condition in PP, and the authors considered this value a reference. The authors suggested that *w_e_*_,init_ varies considerably among the materials tested. The addition of clay to PP without coupling agents leads to a decrease in *w_e,_*_init_ by more than 75%, depending on the type of coupling agent used. The authors pointed out the quality in clay dispersion could be correlated with *w_e_*_,init_. The nanocomposites with lower micron-scale clay particles proved to be those with the highest *w_e,_*_init_. However, such a correlation could not be established between *w_e,_*_init,_ and surface density in sub-micron-scale clay particles. So, the authors underlined that the reinforcement or toughening effects are related to the nanoscale particles, as concluded from tensile results analysis. The authors also analyzed the fracture surfaces. They observed the fracture occurred by void initiating at larger clay particles, followed by void growth and coalescence as the surrounding matrix stretched into ligaments. Thus, toughness improvements were attributed to higher voiding stresses and improved matrix resistance attributed to finer, more oriented clay nanoparticles.

More recently, other authors applied the EWF technique on PP nanocomposites reinforced with Boehmite clay [267,268]. Pedrazzoli et al. [267] studied the influence of boehmite nanoparticles with various surface treatments on the mechanical properties and fracture behavior of polypropylene copolymer nanocomposites. The boehmite was used in pristine and surface-treated forms, adding 2.5, 5, and 10 wt.% using a co-rotating twin-screw extruder. The nanocomposites were successively blow molded to produce films with a thickness of about 0.05 mm. The authors performed an extensive morphological, rheological, thermal, and mechanical characterization. The results obtained were related to fracture behavior. Thus, the authors found that the specific EWF (*w_e_*) of PP increased after incorporating boehmite. Since the crystalline morphology did not show significant changes, the reinforcing effect was mainly attributed to the nano-reinforcement. However, the increase in the filler content induces a reduction in the *w_e_* values, resulting from an excessive filler content that contributes to the agglomeration of the nanoparticles. According to the authors, this effect was also reflected in the reduction in yield stress.

Regarding the treated boehmite, the nanocomposites showed an increase in resistance with low reinforcement content. However, *w_e_* did not show significant variations and even decreased. The authors declared that these results are in accordance to observed by other authors [237] regarding the direct proportion between *w_e_* and e0∙σ_y_, where e0 is the ordinate intercept of extension-at-break versus ligament length linear regression plots. In addition, the authors also reported lower *βw_p_* values when compared with unfilled PP. Therefore, the nanofillers do not influence the dissipative plastic work.

Turcsán et al. [268] used the EWF procedure to determine the fracture toughness of poly (propylene-blockethylene) (EPBC)-based nanocomposites with different boehmite content (0.5, 1, 2.5, 5 wt.%) using a twin-screw extruder. The nanocomposites were compression molded to obtain sheets of 0.5 mm thickness. The degree and quality of the dispersion of the boehmite reinforcement were analyzed using transmission electron microscopy, revealing good distribution and particle agglomerations. The manuscript detailed the efforts to carry out the energy partition method, even though the authors observed that the yield is not instantaneous but develops over time due to the blunting effect. Consequently, the authors indicated that yielding and necking/tearing processes are somewhat superimposed, and only the maximum load may serve for their separation. The results show that a maximum value of *w_e_* is reached with 1 wt.% of boehmite content. Higher concentrations lead to a reducing *w_e_* parameter, similar to those obtained by the unreinforced EPBC.

Similarly, *βw_p_* decreases as the reinforcement content increases. The authors separated the *w_p_* values from the shape factor *β*, considering the plastic zone as an ellipse. The results indicate a marked reduction in *w_p_* with the filler content, while *β* remained practically constant, with values very similar to the unfilled EPBC. The authors concluded the dispersion and the filler content have a significant impact on the necking section. The boehmite nanoparticles did not act as a reinforcement, but it hinders the initiation and growth of cracks.

Haghnegahdar et al. [277] evaluated the fracture toughness of polypropylene (PP)/ethylene propylene diene monomer (EPDM)/graphene nanocomposites prepared by melt mixing process via an internal mixer. The authors used multi-layer graphene (MLG) and few-layer graphene (FLG) with 0.5 wt.% as reinforcement. The DDENT specimens were obtained by compression molding with a thickness of 1 mm. The essential work of fracture (EWF) method was used to describe the deformation mechanism and fracture toughness behavior of PP/EPDM and its nanocomposites based on FLG or MLG in two systems (vulcanized and un-vulcanized). The results indicate that *w_e_* and *βw_p_* of the PP/EPDM fracture enhanced by dynamic vulcanization and also FLG platelets were more efficient in relation to MLG platelets. The SEM micrographs from the fractured surface and subsurface of DENT specimens in the EWF test revealed that the fracture mechanism of PP/EPDM and its nanocomposites were changed by dynamic vulcanization. In the case of vulcanized samples, the fracture toughness mechanism originated from creating cavitation in the dispersed phase, which caused the formation of nanovoids inside the EPDM droplets. This phenomenon caused the dilatation of shear bands and the development of shear yielding of PP matrix. However, in un-vulcanized specimens, the formation of that dilatation band was more prominent due to debonding of rubber droplets from the surrounding PP matrix. In the PP/EPDM nanocomposites, graphene type played a different role in the fracture toughness mechanism. The MLG acted as a suitable domain for crack initiation, which decreased the essential work of fracture. Therefore, FLG platelets hindered the crack path and prevented the crack propagation, leading to increased plastic work of fracture.

**Figure 24:**
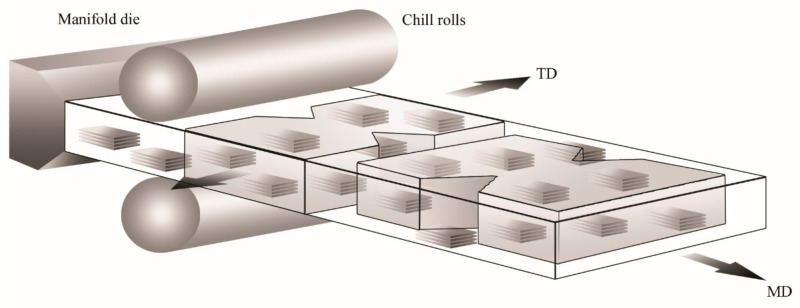
Schematic representation of the cast film extrusion and the orientation of DDENT specimens in MD and TD. The figure represents the clay particle orientation and its influence on the fracture behavior.

Franco-Urquiza et al. [260] evaluated the influence of oriented platelets for intercalated and exfoliated morphologies on the fracture parameters of EVOH/oMMT films through transmission electron microscopy (TEM). The observations revealed that the longitudinal axis of the whole particles was disposed parallel to the melt direction (MD). In order to evaluate the effect of the particle orientation on the fracture parameters, DDENT specimens were cut in the melt and transverse directions (TD). Considering this, the FPZ was perpendicular or parallel to the length of the whole particles, as presented in Figure 24.

The fracture surfaces of DDENT specimens in both configurations (MD and TD) showed distinct behavior related to the orientation of the oMMT particles. According to the SEM observations, the FPZ of the polymer film containing 2.5 wt.% of oMMT in MD showed a necking ligament section notoriously lower than the nanocomposites tested in TD. At higher magnifications, the fracture surfaces of the nanocomposites showed a high level of fibrillation, being more evident for the DDENT specimens tested in MD than in TD, which was attributed to the orientation of the clay particles within the polymer matrix that hinder the plastic flow during the fracture process. Regarding the fracture parameters, the results show that the clay particles act as effective reinforcement since the toughness increased in both configurations (MD and TD). However, *βw_p_* did not show an evident tendency, and it was necessary to calculate the shape factor. Thus, *β* increased with the clay content in MD and decreased in TD because the longest longitudinal axis was oriented parallel to the crack propagation, promoting lower stress transfer than in MD. On the other hand, *w_p_* decreased for both configurations (MD and TD) because the clay particles restrict the plastic flow during the fracture process.

**Figure 25:**
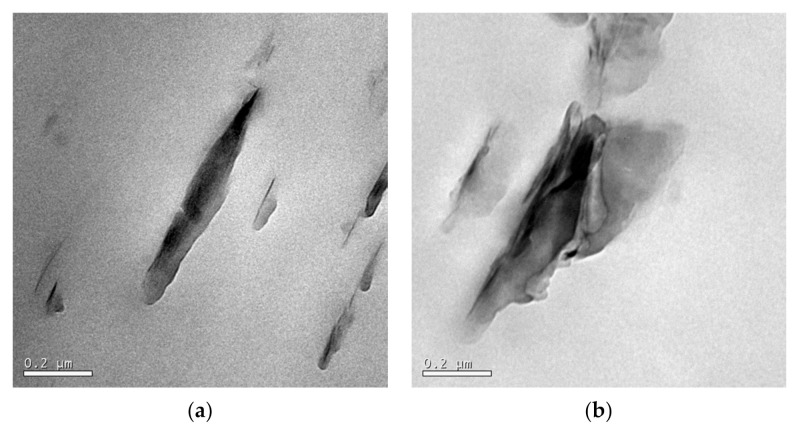
TEM micrographs showing distinct morphologies: (**a**) laminar, (**b**) rolled. The micrographs intend to represent the effect of molecular compatibility between polymer and organomodified clays.

Many commercial clay minerals contain organic modifiers. This modification favors molecular diffusion as long as there is an affinity between the organic modifier and the polymer chains. Franco-Urquiza et al. [281] demonstrated that molecular compatibility could dramatically influence the morphology of clay sheets (Figure 25) and their degree of exfoliation. They used two distinct organo-montmorillonite (oMMT) clays in ethylene-vinyl alcohol (EVOH) copolymer film. The affinity between EVOH and the modifier within oMMT resulted in intercalated morphology because of well-dispersed and oriented platelets due to the sliding during processing (Figure 25a). The low affinity between EVOH and organoclay led to immiscible systems with tactoids by folding the platelets from the clay stacks (Figure 25b). Both morphologies altered the mechanical parameters and the fracture behavior in different ways. The specific essential fracture work (*w_e_*) was influenced by the clay content and crystallinity in both cases. The shape factor increased with the clay content, which implies that the oMMT transfers the stresses for affinity nanocomposites. In inadequate affinity systems, clay particles promoted plastic deformation at low stress levels, according to the values obtained from the specific plastic work (*w_p_*) and observations by SEM. On the other hand, it was observed that the degree of exfoliation decreased as the clay content increased, promoting the development of agglomerated particles.

Block copolymers represent a particular class of self-assembled nanostructured materials, the structure and size of whose morphology can be controlled by molecular architecture, molecular weight, and composition. Martin Ganß et al. [261] studied the influence of oligostyrene-modified montmorillonite (os-MMT) on the morphological, mechanical, and fracture behavior of styrene-butadiene-based block copolymer. They performed the EWF tests to evaluate the crack propagation using a pseudo-single specimen. TEM observations detected a high degree of exfoliation of MMT against well-distributed tactoids with gallery spacings of approximately 10 nm. The authors observed a maximum in the resistance to crack propagation at 3 wt.% of os-MMT accompanied by a change in deformation mechanism from homogenous plastic flow (drawing and micro-necking of microdomains) in the block copolymer nanocomposite (BCP-NC) with 0 to 1 wt.% of os-MMT to a craze-like deformation (microvoid formation and stretching of polymer fibrils) for the BCP-NC with 3 to 5 wt.% of os-MMT. Furthermore, above 5 wt.% os-MMT content a tough-to-brittle type transition attributed to the micro-sized aggregates combined with increased content of rigid PS phase controlling the fracture process BCP-NC with 10 wt.% nanofillers could be ascertained. The authors made an in-depth analysis of the fracture surface, and carried out the correlations between the structural attributes, mechanical behavior and the crack toughness behavior of the developed nanocomposites. They highlighted that the crack initiation behavior, characterized by the magnitude of the specific essential fracture work *w_e_*, changes from being dominated by the polymeric matrix to being dominated by the nano-reinforcement close to 1 wt.%. By increasing the filler content, (3–5 wt.%), the intercalated silicate platelets favored a higher fraction of nano-confined layers in morphology and therefore improved the overall hard phase content, leading to a three-fold reduction in the value of *w_e_*. However, resistance to crack propagation *βw_p_* increases. The authors attributed these results to a change in the deformation mechanism of the homogeneous plastic flow. Finally, the authors observed that higher concentrations of reinforcement, above 5 wt.%, the transition from toughness to brittle observed in the fracture behavior was attributed to the aggregates micrometers in size and the increase in the hard phase content.

Polyamides contain a repeated CO-NH group within the chain. They are well-known engineering thermoplastic materials widely used in industrial applications because of their remarkable mechanical and thermal properties. However, moisture absorption is a relevant limitation. The incorporation of nanometer-scale reinforcement improves the stiffness and toughness of PA.

Baldi et al. [277] evaluated the rubber toughening of polyamide 6 (PA6)/layered-silicate nanocomposites. Nanocomposites with different reinforcing contents were prepared by melt intercalation. For this, a 20 wt.% masterbatch was extruded, which was then diluted in PA6 matrix using a co-rotating twin-screw extruder to obtain concentrations of 4 and 6 wt.%. Three rubber contents were used (0, 5, and 10% by weight). The authors also evaluated the influence of humidity, analyzing the behavior of the various nanocomposites at three humidity levels: dry, slightly humid, and very humid. The authors found that silicate layers in the PA6 matrix strongly influenced the creep and fracture behavior of the material, increasing the elastic limit and reducing the resistance to fracture, depending on the moisture content. The addition of a rubbery phase increased the fracture strength of the nanocomposites under slightly humid conditions, providing evidence of a hardening effect. At the same time, they did not observe a significant increase in fracture toughness in the materials under dry conditions. The authors concluded that a good balance between stiffness and toughness can be obtained under certain humidity conditions, using a suitable rubber-to-silicate layer content ratio.

There is an extensive literature review regarding evaluating the EWF technique for thermoplastic polymers, especially for polyolefins, as previously indicated. However, there is a wide field of opportunities to test the fracture toughness in thermoplastic natural rubber (TPNR) reinforced with clay minerals. An extensive literature review has been carried out, and just one reference on the fracture behavior of these nanocomposites was found. TPNR is a blended material made from natural rubber (NR) and a thermoplastic, such as polypropylene, polystyrene, and polyethylenes, which provides intermediate properties between NR and plastics. Currently, TPNR is used for several industrial applications. Ahmad et al. [309] performed the analysis of the mechanical properties and fracture toughness of TPNR nanocomposites. The nanocomposites were prepared in a twin-screw extruder, varying the concentration of the MMT clay content intercalated with octadecylamine. The authors developed two nanoclay dispersion methods. In the first method, called the direct method (DM), the nanoclay was incorporated into the fused TPNR matrix. In the second method, called the indirect method (IDM), the organoclay was pre-mixed in liquid natural rubber (LNR) before mixing in extrusion. The fracture toughness was evaluated through the EWF technique using 1 mm thick sheets. The addition of optimum organoclay content led to a substantial improvement in stiffness. However, the authors note that the addition of organo-clay reduced the total work of fracture w_f_ for both nanocomposites. The EWF results show that incorporating 4 wt.% organo-clay causes a notable reduction in *βw_p_* for IDM nanocomposites compared to the TPNR matrix. In the case of DM nanocomposites, the authors observed the same trend with the decrease in the values of both specific fracture parameters. The authors attributed these trends to the stiff nature of the clay, which prevents plastic deformation from occurring and the constrained mobility of polymer chains in the presence of organo-clay particles.

Polylactic acid, or polylactide (PLA), is a biodegradable, biocompatible, and renewable thermoplastic polyester, which is mainly derived from corn starch. PLA is classified as an aliphatic polyester because of the ester bonds that connect the monomer units. This bio thermoplastic is the most comprehensively explored biodegradable and renewable thermoplastic polyester. However, uncontrolled degradation, poor thermal properties, and brittleness are the significant drawbacks of PLA. Consequently, PLA nanocomposite systems have been widely exploited to address some of the shortcomings.

Arroyo et al. [308] investigated the processing and fracture behavior of PLA/thermoplastic starch/montmorillonite nanocomposites. Thermoplastic wheat starch (TPS) and PLA were combined with a non-organic MMT clay, using a twin-screw extrusion process to investigate the structure and properties of these nanocomposites. The method of incorporation and gelatinization of starch was based on the technique developed by Rodríguez-González and collaborators [312].

The authors also paid particular attention to moisture content to assess the degree of intercalation/exfoliation of MMT platelets. The authors evaluated the mechanical properties of these biodegradable nanocomposites and their fracture behavior using DDENT specimens and following the EWF protocol. The authors made two relevant observations. The first is that the data obtained met the criteria of self-similarity. Yield stress increased with the ligament length and the general shape of the stress–strain curves was similar. The second general observation is that all the investigated materials exhibited relatively brittle behavior. The initiation energy was significantly higher than the propagation energy, which resulted in both polymers showing a relatively small plastic term, expressed at a low coefficient of linearity. This observation allows the authors to express their questions about whether the plane stress conditions were fully developed during the EWF tests.

The results indicate that PLAg (PLA with maleic anhydride grafted) has lower *w_e_*_,ini_ and *w_e_*_,tot_ values than neat PLA, which the authors attributed to a lower molecular weight. The authors also found that the presence of TPS in PLA led to a considerable reduction in *w_e_*_,ini_ and *w_e_*_,tot_ while *βw_p_* increased dramatically. However, the presence of TPS in PLAg did not lead to such a reduction in *w_e_*_,ini_ and *w_e_*_,tot_ parameters but did lead to a similar increase in *βw_p_* values.

The clay particles showed a higher affinity with the TPS phase. The clay in the TPS phase remains in it, while the clay in the PLA phase migrates to the interface of the blend or even crosses the interface to the TPS phase. The addition of MMT clay increased the tensile modulus of the materials. This observation was most noticeable in the blend with higher TPS content because the tensile modulus of TPS is much lower than PLA. Fracture toughness and elongation at break decreased with the addition of clay. The authors concluded that clay at the interface of the mixtures could reduce the interaction between the PLA and TPS phases in compatibilized mixtures, resulting in a lower stress transfer from the PLA matrix to the disperse TPS phase.

Maspoch et al. [224] employed the EWF methodology to evaluate the ductile–brittle transition behavior of biodegradable polymers. The authors confirmed that the EWF was successfully applied in the organo-modified montmorillonite-based poly(lactic acid) films (PLA/oMMT). In order to evaluate the fracture behavior, the PLA/oMMT films were subjected to a de-aging process. According to differential scanning calorimetry (DSC) results, physical aging at 30 °C of PLA/o-MMT samples exhibited slower enthalpy relaxation kinetics than the neat PLA, which pointed to the toughening mechanisms promoted by oMMT. Similarly, Rodríguez et al. [306] used the PLA/oMMT to evaluate the specific essential work of fracture and compare it with the small punch test (SPT) methodology. The results show that the SPT is an effective tool for the mechanical characterization of these materials because it is possible to perform, from one test with a small sample, the mechanical characterization, and the fracture toughness. The straightforwardness of both the testing procedure and the preparation of the specimens facilitates the rapid characterization. However, the results also reveal that the SPT test is susceptible to prior defects and the oMMT content, allowing the applicability of the EWF method for fracture characterization in ductile materials.

## 4. Conclusions

This work compiles a relevant amount of information on the influence of clay minerals on the fracture behavior of ductile nanocomposites using the EWF technique. From the literature collected, some conclusions can be highlighted:

1. Although clay minerals are commonly used in the development of clay-based polymer nanocomposites, the montmorillonite clay is widely studied. Therefore, there is a wide field of research to explore with the rest of the phyllosilicates.
2. Most clay-based polymer nanocomposites are processed by twin-screw extrusion, leaving aside the use of single-screw extruders. Although the shear stress generated by the twin-screw technology is very efficient in the exfoliation and dispersion of nanoclays (especially polyamides), it promotes the degradation of both the polymer matrix and the organic compounds contained in the modified clays. However, references about processing-induced degradation and its effect on the fracture toughness of polymer nanocomposites were not found.
3. The EWF approach allows the evaluation of the fracture behavior of ductile polymers. However, most of the literature focuses on polyolefins and polyamides. One field that is currently being explored is the study of toughness in bio-based polymer nanocomposites. PLA is the most widely used material, but there are many research opportunities in evaluating the fracture behavior of green polymer nanocomposites.

## Figures and Tables

**Table 1:** Classification of clay minerals.

Structure	Dioctahedral	Trioctahedral
T:O	Kaolinite group [10,11,12,13,14,15,16,17,18,19,20,21,22,23,24,25,26]	Serpentine group [27,28,29]
	Pyrophyllite	Talc
T:O:T	Smectite group [30,31,32,33,34,35,36,37,38,39,40,41,42,43,44]	
	Montmorillonite [45,46,47,48,49,50,51,52,53,54,55,56,57,58,59,60,61,62,63,64,65,66,67,68,69,70,71,72,73,74,75,76,77]	Saponite
	Beidellite	Hectorite
	Nontronite	Stevensite
	Vermiculite group [14,78,79,80,81,82]	
	Illite	
	Mica group [81,83,84,85,86,87,88,89,90,91,92,93,94,95,96,97,98,99,100,101,102,103,104,105,106,107,108,109,110,111,112,113,114,115,116,117,118,119,120,121,122,123,124,125,126]	
	Muscovite	Biotite
	Paragonite	Phlogopite
		Lepidolite
T:O:T:o	Chlorite group [127,128,129,130,131,132,133,134,135]	
	Paligorskite	Sepiolite

**Table 2:** Mechanical properties for clay-based polymer nanocomposites.

Mechanical Test	References
Tensile	[3,157,158,159,160,161,162,163,164,165,166,167,168,169,170,171,172,173,174,175,176,177,178,179,180,181,182,183,184,185,186,187]
Compression	[164,181]
Bending	[188,189,190,191,192,193]

**Table 3:** Issues related to the validity and evaluation of the EWF technique.

Issues	Evaluation	References
Tested specimens	DDENT specimen dimensions. Use of a video extensometer. Notch sharpening. Ligament lengths	[223,224,225,226]
Test conditions	Test rate. Test temperature EWF in mode III.	[208,217,227,228,229,230,231,232,233,234]
Analysis of the results	Energy partitioning. Other geometries for *β*. *w_e_*-J_0_ relationship	[235,236,237,238,239,240,241,242,243,244]

**Table 4:** Summary of the literature regarding to the EWF method applied to polymer nanocomposites.

Polymer	Filler	References
Polypropylene	Montmorillonite clay	[232,259,262,263,264,265,266]
	Boehmite clay	[267,268,269]
	mica	[270]
	Innosilicate	[271]
	Carbon nanotubes	[272,273,274,275,276]
	Graphene	[277]
	Other nanoparticles	[257,278,279]
Polyethylene	Montmorillonite clay	[260,280,281,282]
	Boehmite	[283]
	Mg–Al layered double hydroxide (LDH)	[284]
	Other nanoparticles	[285,286,287,288,289]
Styrene	Montmorillonite clay	[290,261]
	Carbon nanotubes	[291,292]
Polyamide	Montmorillonite clay	[227,293,294,295,296,297,298,299]
	Other nanoparticles	[300,301,302,303]
Polycarbonate	Carbon nanotubes	[304,305]
Biodegradable polymers (PLA and others)	Monmorillonite clay	[224,306,307,308]
	Boehmite	[307]
Thermoplastic natural rubber (TPNR)	Montmorillonite clay	[309]

## Data Availability

This study did not report any data.
